# Editorial: Mental health in a prison setting: Implementation and practice (mhPIP)

**DOI:** 10.3389/fpsyt.2023.1150679

**Published:** 2023-02-22

**Authors:** Emilio B. L. Ovuga, Dickens H. Akena

**Affiliations:** ^1^Department of Mental Health, Gulu University, Gulu, Uganda; ^2^International Institute of Medicine and Science, Rancho Mirage, CA, United States; ^3^Bomvitae Agro Industries Limited (BAIL), Kampala, Uganda; ^4^Department of Psychiatry, Makerere University College of Health Sciences, Makerere University, Kampala, Uganda

**Keywords:** mental health, psychiatry, prison, women offenders, suicide, deliberate self-injury, 9 correctional institutions, juvenile offenders

The practice and delivery of mental health services in the prison setting present unique challenges. While the goal of correctional institutions is to transform and rehabilitate offenders into law-abiding members of society, the goal of psychiatry is to restore the positive mental health of mentally ill offenders. The nature and magnitude of crimes are as varied as the offenders. Moreover, the nature of interactions between therapists and prison inmates may be overlooked, and not systematically used in treatment of these individuals (1, 2). Therefore, it may be easy to inadequately prepare prison inmates with multiple needs (3, 4) for their return to their environments (5).

The chapter from northern Russia highlights the importance of correctional workers and mental health professionals being aware of the potential risk of suicide and non-suicidal self-destructive behavior among young prison inmates (Koposov et al.). Suicidal behavior is (a) always present among care seekers and inmates of correctional institutions and (b) suicidal urges and acts can be predicted and identified long before they occur in most cases. A chapter from the Female Prison in Hunan Province, China, revealed that 9.47% of women incarcerated met the criterion for post-traumatic stress disorder (PTSD) on admission to the prison (Zhong et al.). This finding should remind us that, amongst individuals admitted to prison, a number of them enter the prison system with one or more pre-existing mental disorders. It therefore makes sense to screen first-time detainees in a correctional institution for post-traumatic stress disorder. Another study on women incarcerated for the first time in Geneva Hospital in Switzerland revealed a significantly higher prevalence rate of mental disorders; 16.9% compared with 1.3% among those detained on a repeat occasion (D'Orta et al.). A study in Geneva, Switzerland, revealed an extremely high prevalence (82.6%) of two or more psychiatric disorders in incarcerated youths compared with those not incarcerated. These results showed that incarcerated youths do suffer from one or more unrecognized mental disorders (Heller et al.). However, a longitudinal study on correctional officer recruits in Canada reported an extremely low prevalence rate of 4.9% for one or more psychiatric disorders in this group, a finding that clearly deviates from the previously published rate of 54.6% (Easterbrook et al.). The study is a useful pointer to the need for correctional officers to undergo training and meticulous screening, not only for their suitability for employment, but also to detect the presence of poor mental health.

The implications of these studies are: (1) a proportion of offenders who are detained to a correctional center for the first time may present with unrecognized mental disorder, (2) a proportion of these offenders have multiple psychiatric disorders along with a variety of social needs, (3) screening of the mental status of recruits may yield benefits for both future correctional officers and offenders, (4) women present a special vulnerable group for mental disorders and other problems associated with incarceration, (5) the prison system may provide a conducive environment for suicide and non-suicidal self-harm among youths detained for criminal behavior, (6) those who enter the correctional institution are just as human as those outside prison, and (7) more longitudinal studies are recommended in order to improve the quality of correctional care and mental health care provision in the prison setting. Elaborated efforts, covering social needs, mental health problems, and correctional system, are needed.

## Author contributions

EO drafted this editorial. DA reviewed and approved the manuscript for publication. EO and DA accept responsibility for the contents of the manuscript. All authors contributed to the article and approved the submitted version.
